# Variations in the structure and function of the soil fungal communities in the traditional cropping systems from Madeira Island

**DOI:** 10.3389/fmicb.2024.1426957

**Published:** 2024-10-01

**Authors:** Maria Cristina O. Oliveira, Artur Alves, Cátia Fidalgo, José G. R. de Freitas, Miguel A. A. Pinheiro de Carvalho

**Affiliations:** ^1^ISOPlexis Centre of Sustainable Agriculture and Food Technology, University of Madeira, Campus da Penteada, Funchal, Portugal; ^2^ARDITI, Agência Regional para o Desenvolvimento da Investigação, Tecnologia e Inovação, Caminho da Penteada, Funchal, Portugal; ^3^Department of Biology, University of Aveiro, Campus Universitário de Santiago, Aveiro, Portugal; ^4^Department of Biology, CESAM—Centre for Environmental and Marine Studies, University of Aveiro, Campus Universitário de Santiago, Aveiro, Portugal; ^5^Centre for the Research and Technology of Agro-Environmental and Biological Sciences (CITAB), Inov4Agro - Institute for Innovation, Capacity Building and Sustainability of Agri-food Production, University of Trás-os-Montes and Alto Douro, Vila Real, Portugal

**Keywords:** soil mycobiota, microbial communities, soil biodiversity, functional communities, sustainable agriculture

## Abstract

Agricultural soils are responsible for ecological functions and services that include primary production of food, fiber and fuel, nutrient cycling, carbon cycling and storage, water infiltration and purification, among others. Fungi are important drivers of most of those ecosystem services. Given the importance of fungi in agricultural soils, in this study, we aimed to characterize and analyse the changes of the soil fungal communities of three cropping systems from Madeira Island, where family farming is predominant, and investigate the response of fungi and its functional groups to soil physicochemical properties. To achieve that, we sequenced amplicons targeting the internal transcribed spacer 1 (ITS1) of the rRNA region, to analyse soil samples from 18 agrosystems: 6 vineyards (V), 6 banana plantations (B) and 6 vegetable plantations (H). Our results showed that alpha diversity indices of fungal communities are similar in the three cropping systems, but fungal composition and functional aspects varied among them, with more pronounced differences in B. Ascomycota, Basidiomycota, and Mortierellomycota were the main phyla found in the three cropping systems. Agaricomycetes and Sordariomycetes are the predominant classes in B, representing 23.8 and 22.4%, respectively, while Sordariomycetes (27.9%) followed by Eurotiomycetes (12.3%) were the predominant classes in V and Sordariomycetes (39.2%) followed by Tremellomycetes (8.9%) in the H. Saprotrophs are the fungal group showing higher relative abundance in the three cropping systems, followed by plant pathogens. Regarding symbionts, endophytes were highly observed in B, while mycorrhizal fungi was predominant in V and H. The structure of fungal communities was mainly correlated with soil content of P, K, N, Fe, and Cu. In addition, we identified bioindicators for each cropping system, which means that cultivated crops are also drivers of functional groups and the composition of communities. Overall, the three cropping systems favored diversity and growth of taxa that play important roles in soil, which highlights the importance of conservative management practices to maintain a healthy and resilient agrosystem.

## 1 Introduction

World population growth is a challenge for agriculture (Schulte et al., 2014). To meet the growing demand for food, feed, and fiber, production areas have increased substantially in recent decades and farmers have intensified the use of irrigation, fertilizers, pesticides, and mechanical practices, which have high costs for the environment (Schulte et al., 2014; Foley et al., 2011). Crops in high demand worldwide are permanent or cultivated continuously over vast areas for many years (Jiang et al., 2024; Wahome et al., 2023; Liu et al., 2020). These practices lead to soil deterioration that affects plant growth, yield, and food quality (Jiang et al., 2024). However, in many regions of the world, polyculture has traditionally been used and promoted as a good way to preserve soil health (Pinheiro de Carvalho et al., 2022; Guzman et al., 2021).

Madeira Island is an example of these regions. Madeira is the largest inhabited island of the Portuguese Archipelago of Madeira, located in the Atlantic Ocean, between the latitudes 33°10′–32°20′N and longitudes 16°10′–17°20′W and at 630 km west from the coast of North Africa (Ganança et al., 2007). The location and orography of the island define the climate, showing a mixture of Subtropical and Mediterranean climatic elements. This characteristic makes the island ideal for growing a wide variety of vegetables (Pinheiro De Carvalho et al., 2021) and fruit crops, such as bananas and grapevines (Pinheiro de Carvalho et al., 2022), which provide beautiful landscapes that are very attractive to tourism (Macedo et al., 2020; Ribeiro and Silva, 1998). Due to the small size of most agrosystems (farms), below one hectare, the agriculture in Madeira is mainly familiar, based on low input, rotation, intercropping, or pluricultural practices (Pinheiro de Carvalho et al., 2022). However, most of the banana and grapevine agrosystems are under long-term monoculture.

Healthy soil is imperative in agriculture, regardless of the target crop or agricultural practice. Microorganisms are responsible for many processes that are crucial for soil maintenance and quality (Thomson et al., 2015; Zeilinger et al., 2016). Fungi, due to their ability to produce a wide variety of extracellular enzymes, can regulate the balance of carbon and nutrients (Frąc et al., 2018). Besides, fungi can act as biocontrol agents, since they can be antagonists of other organisms that are pests or cause diseases, and can interact with plant components (Zeilinger et al., 2016; Frąc et al., 2018). Fungi are known to be crucial in supporting plant life; mycorrhizal fungi are known to associate with roots of over 90% of plant species and improve the nutrient status of their host (Bonfante and Genre, 2010). Soil environments are not static and the microorganisms in these habitats react to changes in soil conditions (Choudhary et al., 2022). Soil fungal communities are influenced by several factors, including physicochemical conditions, which depend on geological, climatic, and land use factors (Hartmann et al., 2012; Lauber et al., 2008; Yang et al., 2017). While the ecological services of fungi are critical to agriculture and an important aspect of sustainable agriculture under new scenarios of climate change, management practices are likely to result in changes in soil variables, thus impacting fungal communities with costs for important soil processes (Thomson et al., 2015; Frąc et al., 2018; Hartmann and Six, 2023). For example, the employment of standard and persistent agricultural practices in continuous cropping systems of major crops can result in the selection of specific taxa, reducing the abundance of functionally important fungi and increasing pathogens (Pérez-Brandán et al., 2014). Orrù et al. (2021) demonstrated, in a long-term field experiment, that fungal diversity was greater in the plots where no-tillage/rotation was applied, compared to plots where tillage and/or no-rotation was applied. In addition, these authors identified fungal species that are indicators of management practices or associated with specific conditions.

Until recent years, the study of the structure of fungal communities was limited to morphological identification and culturing (Wijayawardene et al., 2021; Janowski and Leski, 2022). High-throughput sequencing provided a culture-independent approach that allowed for a better understanding of the ecology of soil fungi (Frąc et al., 2022). In the field of agriculture, the impact of management practices on fungal communities has gained some attention in recent years. Nevertheless, more research is needed to understand fungal community dynamics and, therefore, how to positively influence the sustainability and productivity of agroecosystems (Frąc et al., 2018).

To the best of our knowledge, the study of fungal communities in agricultural soil on Madeira Island is scarce and does not focus on the diversity of functional groups or the impact of different cropping systems (Pinheiro de Carvalho et al., 2022; Melo and Cardoso, 2008). Therefore, in this study, using amplicon (targeting the internal transcribed spacer 1, ITS1, of the rRNA region) DNA sequencing with Illumina technology, we aim to: (1) characterize and analyse the changes of the fungal communities of three different cropping systems from Madeira Island: vineyards, banana plantations, and vegetables; and (2) investigate the response of fungi and its functional groups to soil physicochemical properties.

## 2 Materials and methods

### 2.1 Study sites and soil samplings

**Figure 1:**
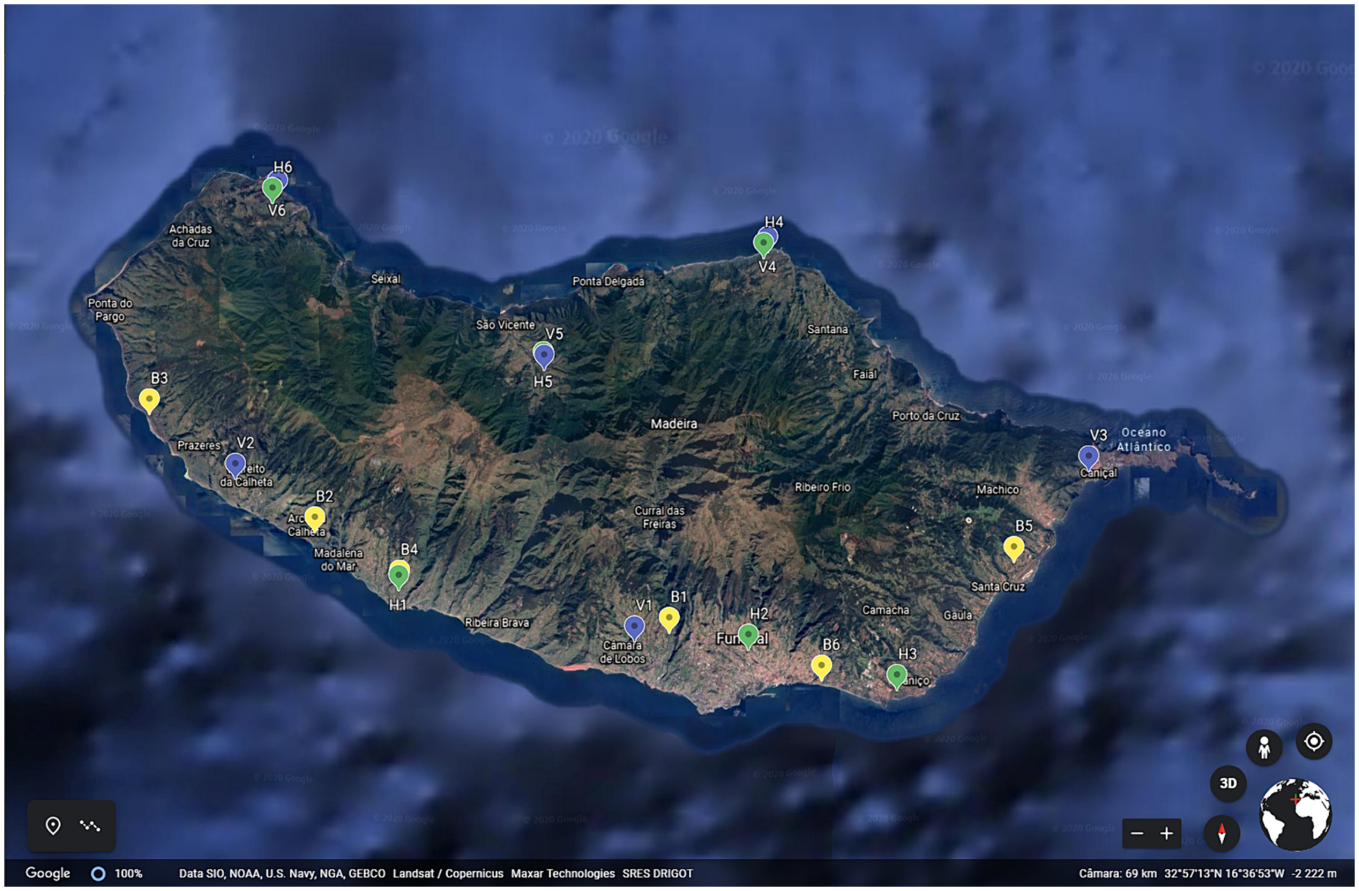
Location of agrosystems under study along Madeira Island. B1–B6 is for banana plantations, V1–V6 is for vineyards and H1–H6 is for vegetable plantations.

Eighteen traditional agrosystems were selected along the island, 6 vineyards (V), 6 banana plantations (B) and 6 vegetables farms (H) (Figure 1). The cropping system adopted in vegetable farming is polyculture, intercropping and/or rotation and in vineyards and banana plantations monoculture. Three vineyards and vegetable farms were in the north and three in the south of the island. However, all banana plantations were located in the south, as the north of the island does not offer suitable conditions for banana crop growing. At the beginning of this study, all agrosystems had been installed for more than 10 years. Soil samples were collected between May and June of 2020, according to Paetz and Wilke (2005), with adaptations. Briefly, soil samples from 15 points, at 10–15 cm deep close to the crops, less than 50 cm apart, were collected along the field, in a zigzag pattern. Samples were bulked to get sample representative of the agrosystem soil. Pooled soil samples (*n* = 18, one per agrosystem) were divided into two parts, one part was immediately transported to the laboratory, distributed in aliquots and stored at −20°C until DNA extraction, the other part was air-dried, ground, and sieved (2 mm) for further analysis to determine pH, organic matter (OM), cation exchange capacity (CEC), degree of saturation (DS), macronutrients (NO_3_-N, NH_4_-N, P, and K) and micronutrients (Ca, Mg, Na, B, Cu, Zn, Mn, and Fe) at the Agricultural Quality Division of Laboratory and Agro-Food Research Services in Camacha, Madeira, Portugal. Determination of pH, OM, CEC, and macronutrients (NO_3_-N, NH_4_-N, P, and K) was done according to Ragonezi et al. (2022). Micronutrients (Ca, Mg, Na, B, Cu, Zn, Mn, and Fe) and DS were determined according to Temminghoff and Houba (2004).

### 2.2 DNA extraction and purification

Total genomic DNA extraction was adapted from Yeates et al. (1998). Extraction buffer (400 μL of 100 mM Tris–HCl, pH 8.0; 100 mM sodium EDTA, pH 8.0; 1.5 M NaCl) was added to 250 mg (dry weight) of soil in a tube with glass beads (≤106 μm, acid-washed; Sigma-Aldrich, United States) and vortexed for 10 min. Sodium dodecyl sulfate (SDS) was added (25 μL; 10%) and samples were mixed for 5 s. Samples were incubated at 65°C for 1 h, centrifuged (16,000 g for 10 min) and the supernatants were transferred to new tubes. The soil pellets were re-extracted with extraction buffer (400 μL), incubated at 65°C for 10 min and centrifuged as above. Supernatants were transferred to the tubes with the previous extraction, then a half-volume of polyethylene glycol (30%)/sodium chloride (1.6 M) was added and incubated at room temperature for 2 h. After centrifugation (16,000 g for 20 min) pellets were resuspended in 140 μL of Tris-EDTA (10 mM Tris–HCl, 1 mM sodium Ethylenediaminetetraacetic acid, pH 8.0). Potassium acetate (7.5 M) was added to a final concentration of 0.5 M. Samples were transferred to the fridge for 5 min and then centrifuged (16,000 g, 30 min). The aqueous phase of supernatants was obtained by addition of phenol/chloroform/isoamyl alcohol (25:24:1) and centrifuged (2,500 g for 15 min). Then, the aqueous phase was precipitated by adding 1.5 volume of absolute ethanol and incubated overnight at −20°C. DNA was pelleted (16,000 g for 30 min) and resuspended in TE (25 μL). The DNA extraction was performed in sextuplicate from each soil sample (*n* = 18*6 = 108) and total DNA was pooled in the end before further analysis (*n* = 18). DNA purity and concentration were measured with NanoDrop^®^ 2000c Spectrophotometer (ThermoFisher Scientific, United States). DNA extracts were purified using ExtractME DNA clean-up kit (Blirt, Poland) and preserved at −20°C.

### 2.3 ITS amplicon sequencing

PCR amplification of ITS 1 region of the fungal rRNA cluster was performed with primers ITS5-1737F (5′ GGAAGTAAAAGTC GTAACAAGG 3′) and ITS2-2043R (5′ GCTGCGTTCTTCATC GATGC 3′) (White et al., 1990). Amplicon cleaning, library preparation and subsequent sequencing was conducted at Novogene (UK), using Illumina Sequencing NovaSeq technology (paired-end reads of 250 nt). Samples were analyzed in duplicate (50 K tags per sample; *n* = 18*2 = 36).

### 2.4 Bioinformatics pipeline

Samples were demultiplexed, and primer sequences were removed with Cutadapt (v3.3) (Martin, 2011). FLASH (v1.2.11) software was used to merge the reads (Magoč and Salzberg, 2011), using default parameters, with exception of fragment length of 300, and a maximum mismatch density of 0.1. Quality control of raw tags was checked using fastp (v0.20.0) software (Chen et al., 2018), where qualified quality phred was ≥Q19 and unqualified percent limit was set to 15%. Vsearch (v2.15.0) software (Rognes et al., 2016) was used to detect and remove chimeric sequences, and obtain the final effective data. The reads were further processed with QIIME2 (v2020.6.0) software (Bolyen et al., 2019). DADA2 (v4.2.0) (Callahan et al., 2016) was used to denoise the reads (with default settings). The length of the final Amplicon Sequence Variants (ASVs) ranged between 200 and 400 bp. ASVs with fewer than 5 reads were removed, and the remaining ASVs were compared with the UNITE database (Abarenkov et al., 2023), using a sklearn-based taxonomy classifier (Bokulich et al., 2018). The abundance of ASVs was normalized using a standard of sequence number corresponding to the sample with least sequences (19,931 high-quality reads). Subsequent analyses of alpha diversity indices and beta diversity were performed based on the output normalized data. The sequence files were submitted to GenBank (https://www.ncbi.nlm.nih.gov/, BioProject: PRJNA1107202, accession: KIGQ00000000).

### 2.5 Statistical analysis

All tests were performed considering an alpha level of significance of 0.05. Alpha-diversity indices, namely Richness (Obs. ASVs), Pielou’s Eveness (E), and Shannon-Wiener (H′) diversity were calculated with QIIME2 software. The rarefaction curves and species accumulation boxplot were drawn. For beta-diversity, samples were compared using the UniFrac distances calculated by QIIME2 software, and the dimensionality reduction maps of non-metric multidimensional scaling (NMDS) drawn by R software (v4.3.1), packages vegan (v2.6-4) (Oksanen et al., 2022) and ggplot2 (v3.4.2) (Wickham, 2016). The significance of community structure differences among groups was calculated, through the analysis of similarity function (ANOSIM R) in QIIME2 software. A heatmap of community composition was plotted in QIIME2 software, after Z-score normalization, and bar charts drawn in Microsoft Excel 2019 (v1808). Linear discriminant analysis Effect Size (LEfSe) analysis was performed by LEfSe software (v1.1.2) (Segata et al., 2011), and linear discriminant analysis (LDA) score threshold was set to 4. FUNGuild database (Nguyen et al., 2016) was used to assign ASVs to potential ecological guilds. In addition, to determine differences among the cropping systems, an ANOVA followed by the Tukey HSD test were performed when data normal distribution was observed. When the data normality was not observed, Kruskal-Wallis and Dunn’s tests with Bonferroni correction were applied. These analyses were performed using the statistical package for the social sciences (SPSS, v27.0) for Windows. The boxplots and bar charts of community composition were drawn using R software (package graphics) and Excel 2019 (v1808), respectively.

Variables of physicochemical properties were tested for normality and the ones not following a normal distribution were transformed using R, package bestNormalize (Peterson, 2021; Peterson and Cavanaugh, 2020), to draw the map for Principal Component Analysis (PCA), using R, packages stats, ggplot2 and ggfortify (Tang et al., 2016). The multiple correlations analysis was performed in SPSS, using the Pearson coefficient or, when normality was not observed, Spearman’s correlation coefficient.

## 3 Results

### 3.1 Impact of the cropping system on fungal abundance and community diversity

The sequencing depth of the metabarcoding analysis was sufficient to cover the diversity in all communities, which can be seen in the rarefaction curves of all samples, which nearly reached saturation (Supplementary Figures S1, S2). The lowest value obtained for observed ASVs, Pielou’s Evenness (E) and Shannon’s diversity (H′) was found in a banana plantation (B4) with the average of 404, 0.39, and 3.42, respectively. The highest values were all found in vineyards, with 834 for obs. ASVs (V1), 0.75 for E (V3) and 7.20 for H′ (V3). No significant differences were detected among cropping systems for the alpha-diversity indices (Supplementary Table S1; ANOVA, *p* > 0.05).

**Figure 2:**
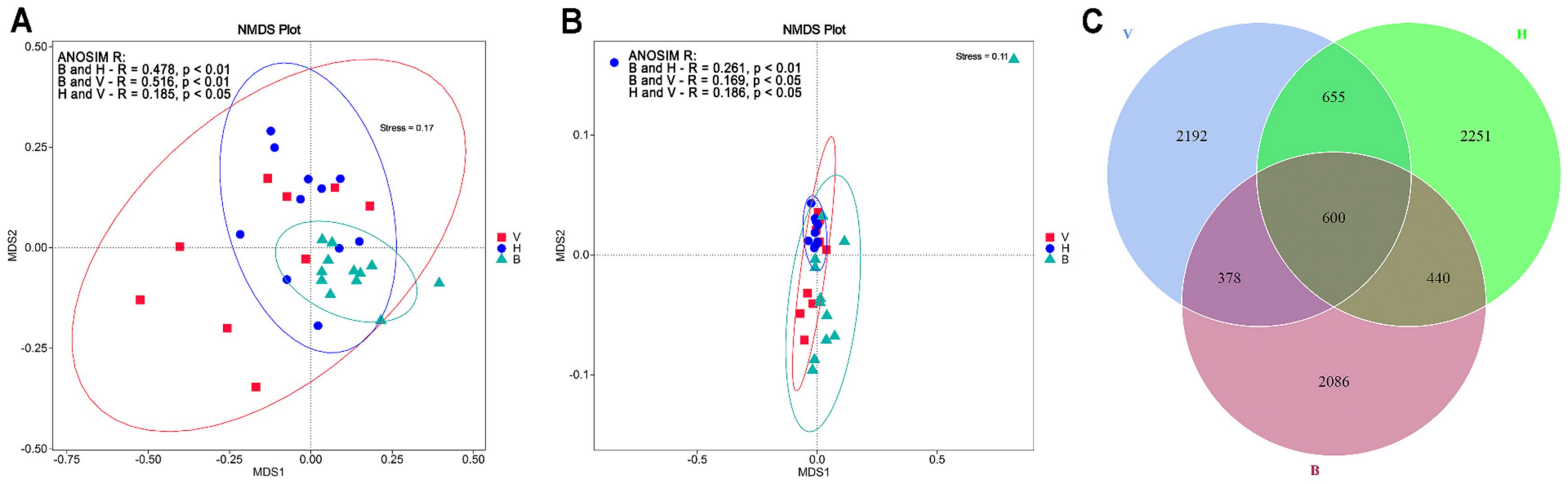
Comparison of the soil fungal communities in the cropping systems (B, V, and H). **(A)** NMDS ordination plot based on unweighted UniFrac distance metric for the 18 sampling sites (in duplicates) and pairwise ANOSIM R test results. **(B)** NMDS based on weighted UniFrac distance metric for the 18 sampling sites (in duplicates) and pairwise ANOSIM R test results. **(C)** Venn diagram showing the number of unique and shared ASVs among and between cropping systems.

The NMDS cluster analysis based on unweighted UniFrac distances showed a great variation among fungal communities between and even within the cropping systems (Figure 2A). The cropping system B showed less dissimilarity between samples. However, they are close to some samples from H and V cropping systems. The NMDS analysis based on weighted UniFrac distance shows that most of the samples from H cluster together and samples from V and B are more dissimilar. Although some of them are close to H cluster (Figure 2B). The unweighted and weighted ANOSIM R test showed that fungal communities from cropping systems were significantly different (*p* < 0.01). Pairwise comparisons for unweighted UniFrac metric (Figure 2A) showed that cropping system B was dissimilar from V and H (R ~ 0.5, *p* < 0.01). The cropping systems V and H were less dissimilar (*R* = 0.19, *p* < 0.05). Pairwise comparisons for weighted UniFrac metric (Figure 2B) showed that the cropping system B was more dissimilar from the H than from the V (R of 0.26 and 0.17, respectively; *p* < 0.05 for all pairwise comparisons). Two samples, one from B4 and one from H6, differed strongly from the others. The Venn diagram (Figure 2C) showed that H sampling sites showed more unique ASVs (2251) followed by V (2192) and B (2086). The V and H are the cropping systems sharing more ASVs (655) and the three cropping systems shared 600 ASVs.

### 3.2 Impact of the cropping system on fungal communities’ composition

**Figure 3:**
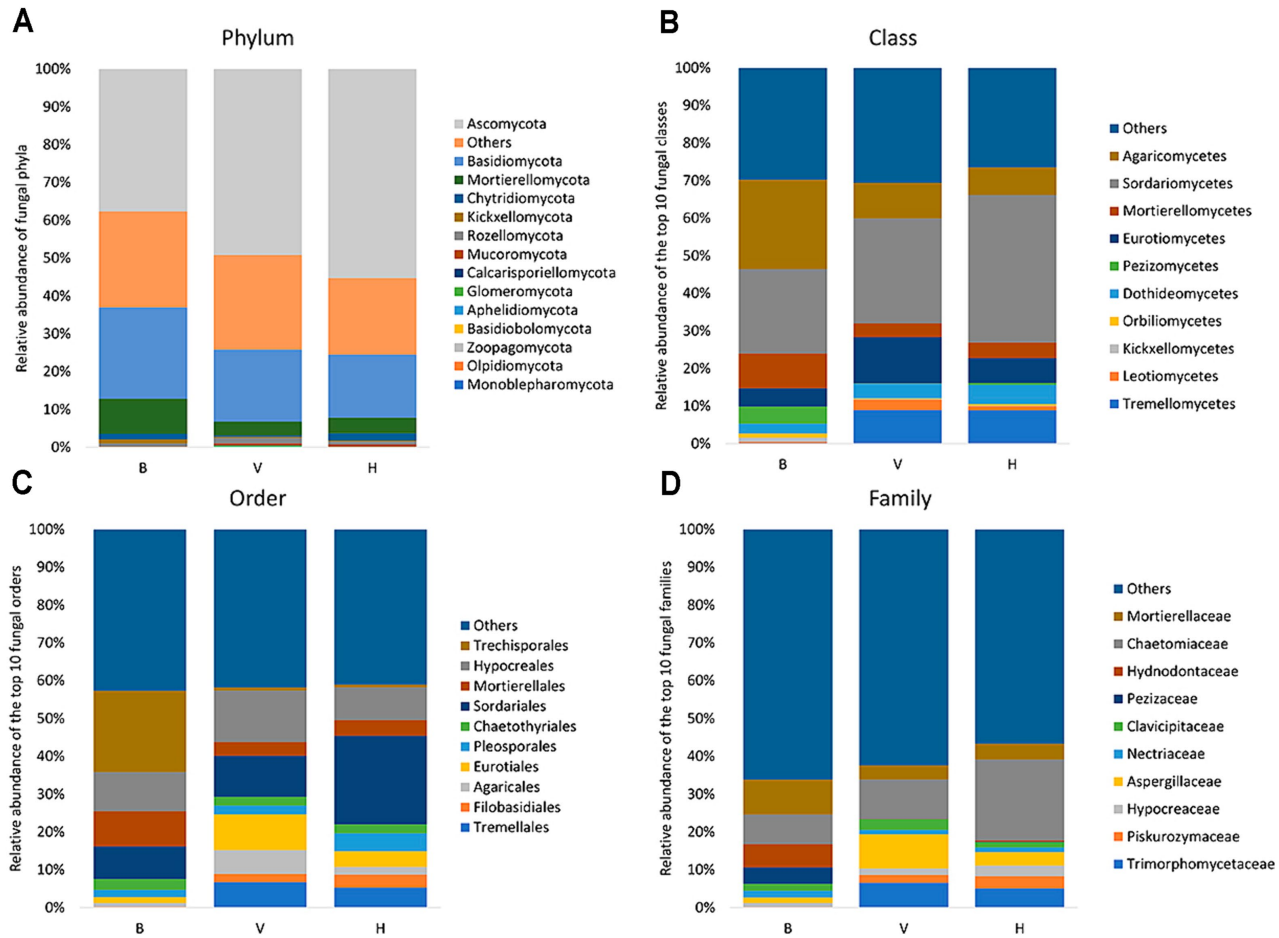
Relative abundance of fungal taxa in each cropping system from phylum to family **(A–D)**. For class, order and family only the 10 most abundant taxa are discriminated in the graphics.

The fungal community composition of the cropping systems is presented in Figure 3. Graphics organized from phylum to family exhibit the most abundant taxa. Fungi from B, V and H belong mainly to phyla Ascomycota, Basidiomycota and Mortierellomycota (Figure 3A), the latter being more abundant in B cropping system. It was not possible to assign phylum-level classification to 20% of total fungi in the three cropping systems. Agaricomycetes and Sordariomycetes are the predominant classes in B, representing 23.8 and 22.4%, respectively, while Sordariomycetes (27.9%) followed by Eurotiomycetes (12.3%) were the predominant classes in V and Sordariomycetes (39.2%) followed by Tremellomycetes (8.9%) in the H (Figure 3B). Trechisporales order represents 21.5% of total fungi in B, and < 1% in V and H (Figure 3C). Mortierellaceae (9.3%), Chaetomiaceae (7.8%) and Hydnodontaceae (6.2%) are the main families in B with a close relative abundance (Figure 3D). However, Mortierellaceae and Hydnodontaceae represents <5% and <1%, respectively in the V and H cropping systems. Instead, Chaetomiaceae (10.5%) and Aspergillaceae (9.1%) are the main families in V, and Chaetomiaceae stands out among fungal community of H cropping system, with 21.4% of relative abundance. So, the analysis below phylum taxonomic level shows that fungal diversity from B stands out from V and H cropping systems.

**Figure 4:**
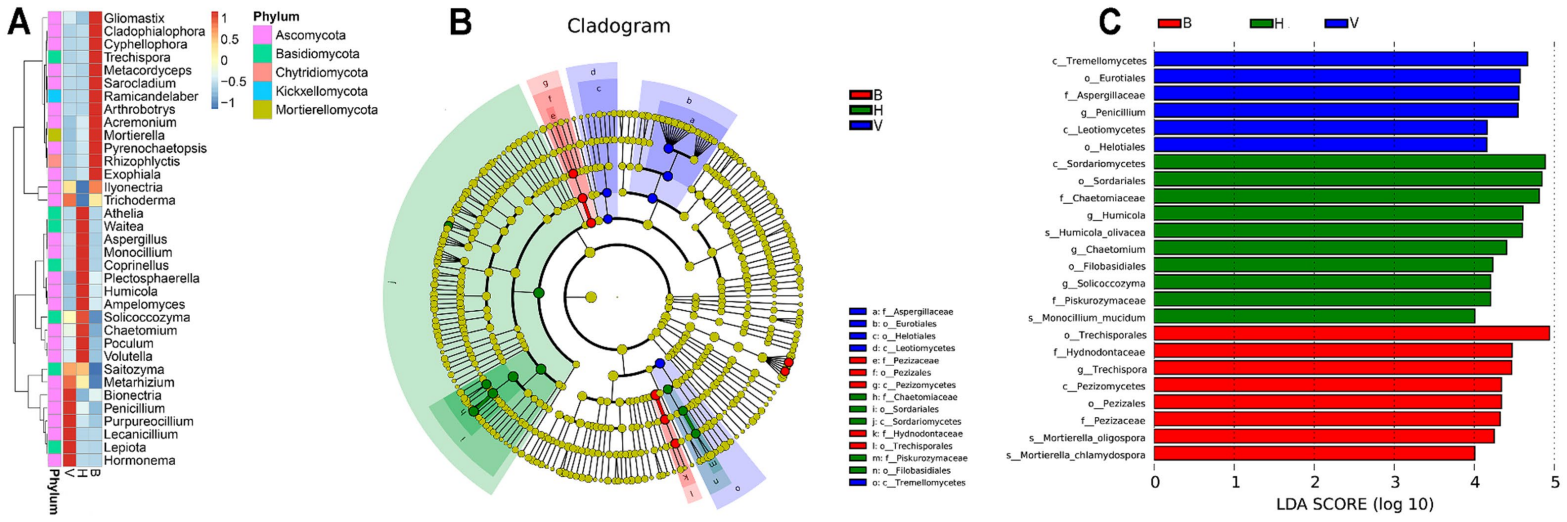
Differences found in fungal community composition of different cropping systems. **(A)** Heatmap of top 35 most abundant genera with the abundance increasing from blue to red, after Z-score normalization. Z values, depicted by colors, indicate the content of fungal species among groups at the genus level. **(B)** Cladogram indicating the polygenetic distribution of fungi. The color of the nodes in the diagram represents the groups that play significant roles in the matching cropping system, while yellow nodes indicate unimportant fungal groups. **(C)** The LDA value distribution histogram shows the taxonomic levels with higher significant differences in abundance in the different cropping systems. Higher scores of LDA represent higher influence of the taxa.

The top 35 of most abundant genera were selected and clustered to draw a heatmap of their abundance across cropping systems (Figure 4A). Each cropping system showed a different set of most abundant genera. It is worth highlighting that the cropping system B holds more genera with higher abundance from 5 different phyla (Ascomycota, Basidiomycota, Chytridiomycota, Kickxellomycota, and Mortierellomycota), while in the V and H, the genera with higher abundance were only from Ascomycota and Basidiomycota. The linear discriminant analysis effect size (LefSe) complements these results, identifying bioindicators responsible for significant differences detected between cropping systems. Significant differences observed at family level or broader levels can be seen in the Figure 4B. The histogram in Figure 4C shows the linear discriminant analysis (LDA) value for potential species or broader taxonomic levels with significant differences in abundance among cropping systems. Twenty-four taxa play an important role in cropping systems differentiation. One genus (*Penicillium*), one family (Aspergilaceae), two orders (Eurotiales and Helotiales) and two classes (Tremellomycetes and Leotiomycetes) were found in significantly higher abundance in V. In cropping system H was found two potential species (*Humicola olivacea* and *Monocillium mucidum*), three genera (*Humicola*, *Chaetomium*, and *Solicoccozyma*), two families (Chaetomiaceae and Piskurozymaceae), two order (Sordariales and Filobasidiales) and one class (Sordariomycetes). Finally, two potential species (*Mortierella oligospora* and *Mortierella chlamydospora*), one genus (*Trechispora*), two families (Pezizaceae), two orders (Trechisporales and Pezizales) and one class (Pezizomycetes) were found to be significantly higher abundance in B.

### 3.3 Impact of the cropping systems on functional fungal communities

**Figure 5:**
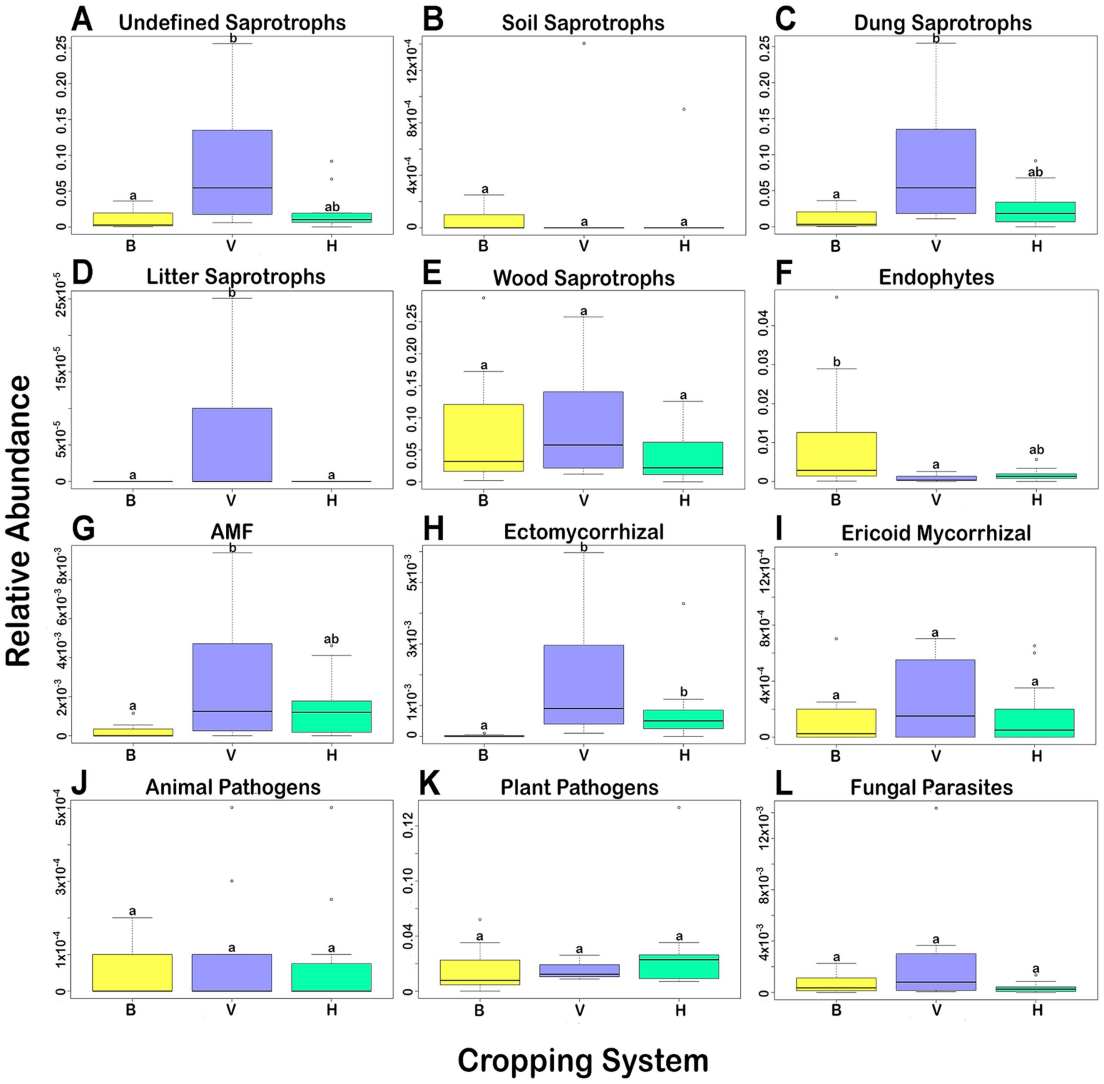
Variation of relative abundance of fungal functional groups in each cropping system. **(A–E)** Saprotrophs; **(F–I)** Symbiotrophs; **(J–L)** Pathotrophs. Boxes with different letters indicate a significant difference among them (Kruskal-Wallis, *p* < 0.05).

Using the database FUNGuild, part of the identified ASVs could be assigned to a potential function in the agrosystems. The variation of the main functional groups in the 3 cropping systems is shown in the Figure 5. Saprotrophs (Figures 5A–E) are the fungal group showing higher relative abundance in the three cropping systems, mainly the unidentified, dung and wood saprotrophs, followed by plant pathogens (Figure 5K) and endophytes (Figure 5F). Significant differences (*p* < 0.05) among the three cropping systems were showed by the Kruskal-Wallis’s test and the post-hoc Dunn’s test with Bonferroni correction. But concerning the unidentified saprotrophs (Figure 5A), dung saprotrophs (Figure 5C), AMF (Figure 5G), and endophytes (Figure 5F), significant differences were observed only between cropping systems B and V. The abundance of ectomycorrhizal fungi was significantly lower in B than in H and V (Figure 5H), and the abundance of litter saprotrophs was significantly higher in V than in B and H (Figure 5D). The other functional groups did not show significant differences among cropping systems.

### 3.4 Soil physicochemical properties in the cropping systems and the effect on soil fungal community structure

**Figure 6:**
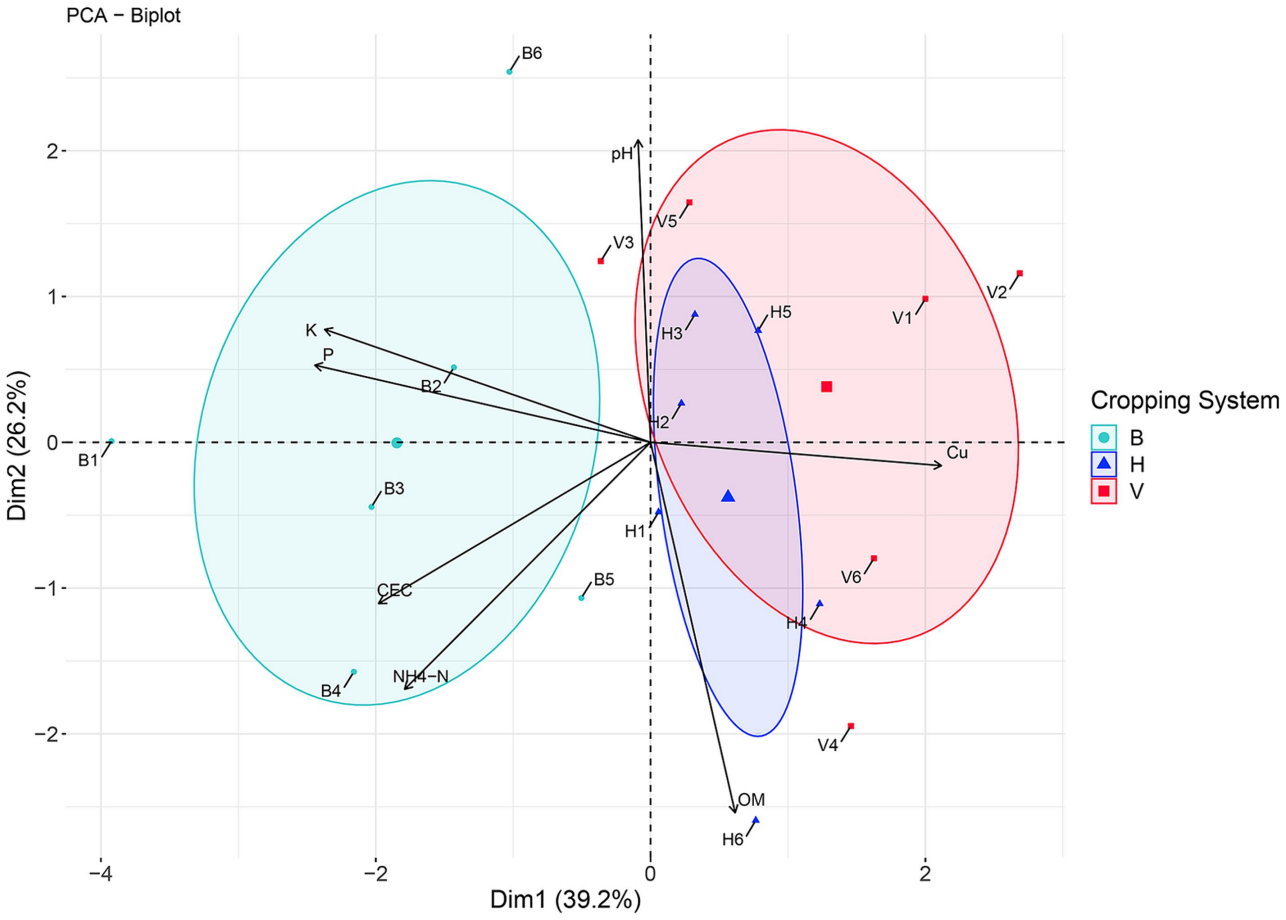
Principal coordinate analysis (PCA) based on soil physicochemical properties in cropping systems B, V, and H.

The PCA explained 65.4% of the similarities among cropping systems based on soil physicochemical properties (Figure 6; Supplementary Table S2). The first and second principal components explained 39.2 and 26.2% of the data variation, respectively, allowing to separate the cropping system B from V and H. The Cu concentration had the higher weight in this separation from V and H cropping systems. However, the variation in CEC and macronutrients NH_4_-N, P and K also had an impact on the separation of cropping system B from H and V, along second PCA axis. The pH and OM variation is well correlated with samples distribution along the first axis. These two parameters distinguished the mean point of V and H, meaning that in average, H had higher concentration in OM and V had higher values of pH.

**Figure 7:**
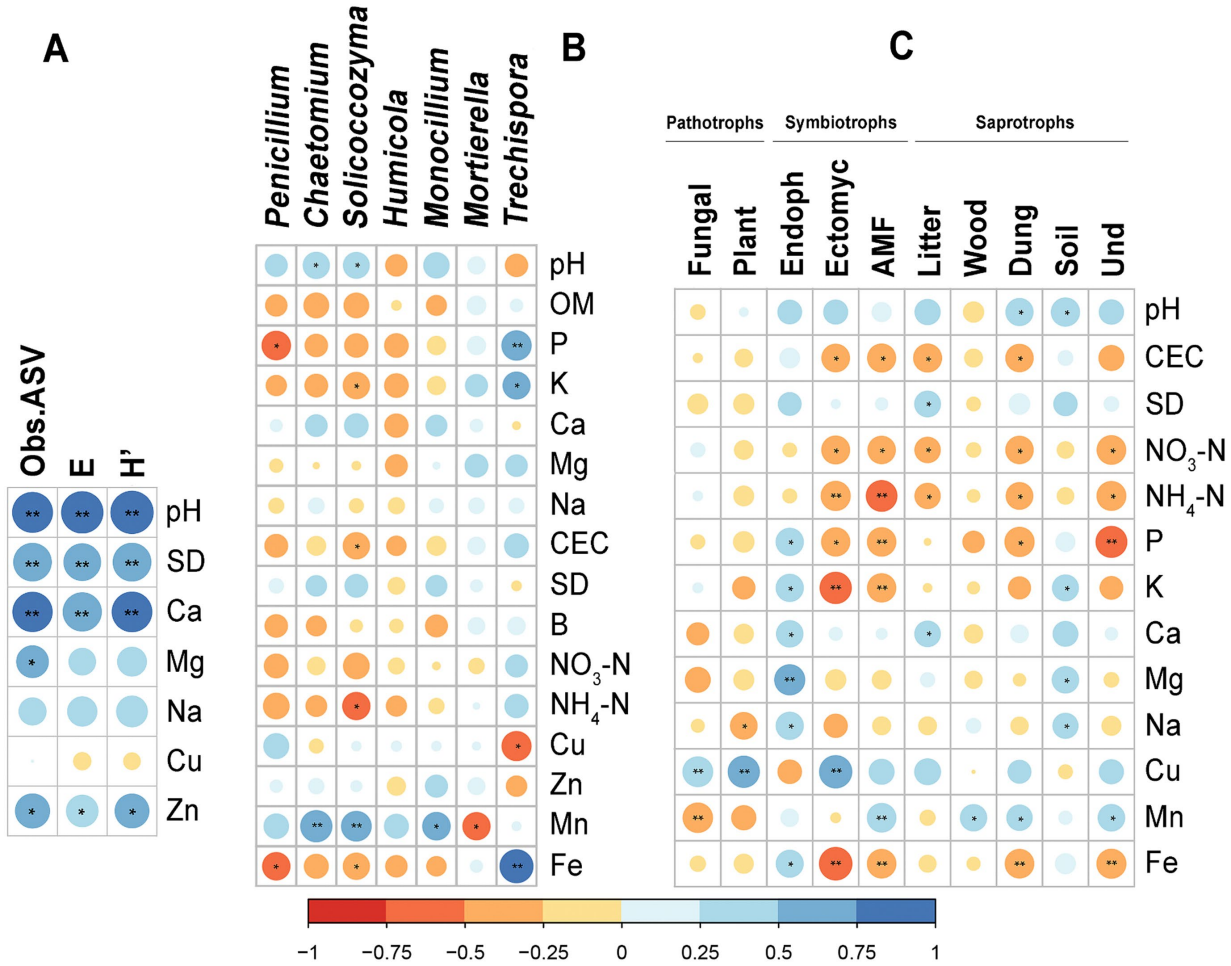
Observed Spearman significant correlations between soil physicochemical properties and **(A)** diversity indices, **(B)** key fungal genus identified as bioindicators in LefSe, **(C)** functional groups. Symbol * for *p* < 0.05; ** for *p* < 0.01. OM, Organic Matter; CEC, Cation Exchange Capacity; SD, Saturation Degree; Obs.ASV, Richness; E, Pielou Evenness; H′, Shannon–Wiener diversity index; Endoph, Endophytes; Ectomyc., Ectomycorrhizal; Und., Undefined.

Soil properties were correlated with diversity indices (Figure 7A). Strong correlations (Spearman, *p* < 0.01) between ObsASVs, E and H′ and pH, SD, and Ca were observed. Significant correlations for micronutrients Mg and Zn (Spearman, *p* < 0.05) were also observed. In addition, it is possible to observe the correlations between soil physicochemical properties and key fungal genus that were identified as bioindicators in LefSe (Figure 7B). Significant correlations were observed for all genus except for *Humicola*. The stronger correlations (Spearman, *p* < 0.01) were observed between *Trechispora* and macronutrient P (*R* = 0.610) and micronutrient Fe (*R* = 0.779) and between *Solicoccozyma* (*R* = 0.626) and *Chaetomium* (*R* = 0.695) with Mn.

Significant positive and negative Spearman correlations were observed between the analyzed soil properties and functional groups determined by FUNGuild (Figure 7C). In this case, macronutrients NO_3_-N, NH_4_-N, P, and K, the micronutrients Mn and Fe and CEC were the soil properties with more significant correlations with functional groups. But other significant correlations were observed between pH, SD, Ca, Mg, Na, and Cu and functional groups. The trophic group of symbionts are the one with more significant correlations with soil properties, as well as the functional group Dung saprotroph. Mycorrhizal fungi and dung saprotrophs were in lower abundance in soils with higher contents of macronutrients Fe, and higher CEC, typically from cropping system B, while endophytes were at highest abundance.

## 4 Discussion

This study represents a first attempt to characterize the soil fungal communities, including its functional groups, of the traditional cropping systems from Madeira Island, namely vineyards, banana, and vegetable plantations. These three cropping systems differ not only in crop, but also in the management practices. Vineyards and banana plantations are in monoculture, while vegetables are under rotation and/or in intercropping. The management practices have influence on soil physicochemical properties (Zalidis et al., 2002), thus changing the environment for soil fungal communities.

In this study we found that fungal community composition and functional groups were more susceptible to the changes across cropping systems, than alpha diversity indices. Values found for diversity (H′) and richness (Obs. ASVs) are similar or even higher to those found in previous studies in vineyards (Gobbi et al., 2022; Pingel et al., 2023; Liang et al., 2019), banana plantations (Wahome et al., 2023; Wang et al., 2022; Jamil et al., 2023) and vegetable plantations (Orrù et al., 2021; Li and Wu, 2018; Wang et al., 2021). Crop rotation is seen as a promising management practice to preserve soil quality (Bai et al., 2018). Even though the analyzed cropping systems have different crops, it was expected that in H, the fungal communities would be more diverse due to the higher plant diversity. However, the alpha-diversity values found for the three cropping systems were similar. Lyu et al. (2020) observed that different vegetables may show opposite effects on Shannon diversity index and richness of fungal communities. Furthermore, the implementation of crop rotation appears to be insufficient to enhance fungal diversity. Other promising management practices should be adopted in combination, such as no tillage (Orrù et al., 2021) or incorporation of residues (Choudhary et al., 2022). The same occurs with intercropping; depending on the consociation, fungal abundance and diversity may or may not be improved (Li and Wu, 2018). The fungal communities from agrosystems of the cropping system B were more similar to each other than in H and V, and the cropping system B is different from H and V. The Venn diagram corroborates with this, showing that B is the cropping system sharing less ASVs with others.

Soil physicochemical properties showed to be determinant in shaping and sizing the fungal communities. Agricultural soils from Madeira island are mainly acidic (Ganança et al., 2007) and liming is frequently used by farmers to increase pH values in order to improve the bioavailability of important nutrients, such as P (Azevedo, 2023; Goulding, 2016). This practice seems to have a positive impact on alpha-diversity indices of fungal communities. Soil pH was pointed as an important factor affecting fungal communities (Wahome et al., 2023; Yang et al., 2017; Zhang et al., 2016; Glassman et al., 2017) and this is in agreement with our results. Special attention should be given to Pielou’s evenness which has been shown to be negatively impacted by low pH of the soil sampling sites. Low fungal evenness may reduce community resistance to environmental changes and this can have serious implications for soil functioning (Anthony et al., 2021). Degree of water saturation also seemed to play an important role in alpha-diversity measurements. In a Mediterranean pine forest, fungal communities showed to be influenced by soil moisture. However, the effect differed among fungal groups (Castaño et al., 2018), arbuscular mycorrhizal fungi were negatively correlated with soil moisture, while free-living fungi were positively correlated. Furthermore, soil moisture also controls the availability of nutrients (Hinsinger, 2001). This can explain why in soils with higher degree of saturation, alpha-diversity indices are improved. Nevertheless, it is worth noting that, in this study, soil samples were collected between May and June, a period with low precipitation and with farmers taking control of irrigation. It is likely that in rainy seasons the behavior of soil fungal communities will be different. For example, in water-saturated soils the fungal activity and the growth of certain taxa was inhibited due to oxygen limitation (Schimel, 2018). A study conducted in a vineyard from Madeira Island, showed that Shannon diversity and Evenness index of fungal communities were significantly lower in winter, the period with the highest precipitation (Pinheiro de Carvalho et al., 2022). Other soil properties are in the base of this process, such as soil texture and structure and the size of the pores (Hartmann and Six, 2023; Samaddar et al., 1834) and should be taken into account when analysing the impact of soil physicochemical properties on soil fungal communities.

In this study, cropping system B was more differentiated from the other cropping systems, especially considering class and lower taxonomic levels. For example, while the three dominant phyla are the same for the three cropping systems (Ascomycota > Basidiomycota > Mortierellomycota), concerning the classes, there are more Agaricomycetes and Mortierellomycetes and less Sordariomycetes in B than in V and H, reflecting the 10 most abundant taxa in the below taxonomic levels. Furthermore, the class Tremellomycetes is almost inexistent in B. Ascomycota and Basidiomycota are predominant phyla in agricultural soils (Choudhary et al., 2022; Wang et al., 2022; Li and Wu, 2018; Wang et al., 2023). Some of these studies also showed high relative abundance of Zygomycota under certain conditions, a phylum where Mortierellomycota was formerly included. The class Tremellomycetes, in H and V cropping systems, include yeasts of the genera *Saitozyma* and *Solicoccozyma*. The genus *Solicoccozyma* was identified as a bioindicator for cropping system H and was found to have correlations with soil physicochemical properties. Despite this, studies demonstrate that the soil yeasts distribution seems to be determined mainly by biotic factors, such as plant, insect and fungal hosts or vectors. Due to yeasts adaptation capacity they can survive in a wide range of environmental conditions (Yurkov, 2018).

Agaricomycetes is a highly diverse class and most of the members live as saprotrophs, plant pathogens or ectomycorrhiza (Naranjo-Ortiz and Gabaldón, 2019). Trechisporales is the most abundant order of Agaricomycetes found in cropping system B, and *Trechispora* was found to be a bioindicator for this sampling group. The genus *Trechispora* is considered as a saprotroph (Hibbett et al., 2014) and is also assumed as wood saprotroph (Nguyen et al., 2016). However, some studies also associate members of this genus to mycorrhizal fungi (Dunham et al., 2007; Vanegas-León et al., 2019; Vohník et al., 2012). The high levels of Fe, P, and K and low levels of Cu seems to be associated to the abundance of *Trechispora*. Two potential species of *Mortierella*, class of Mortierellomycetes, were also found as bioindicators for cropping system B. This genus is classified as saprotroph in FUNGuild database (Nguyen et al., 2016). Due to its ability to degrade organic compounds, such as cellulose (Gawas-Sakhalkar and Singh, 2011) and chitin (De Tender et al., 2019), efficiently solubilize phosphate from soil minerals through pH decreasing (Osorio and Habte, 2013), and other features that can be useful in agriculture (Ozimek and Hanaka, 2021), the *Mortierella* species are being studied as Plant-Growth Promoting Fungi (Zhang et al., 2011; Zhang et al., 2020; Wani et al., 2017).

Cropping systems H and V differ the most at the order level and downwards taxonomic levels. The genera *Chaetomium* and *Humicola*, from the family Chaetomiaceae, were both identified as bioindicators for cropping system H. Members of these two genera are saprotrophic and commonly found in soil and organic compost (Soytong et al., 2001; Ko et al., 2011). Some species have the potential to be used in agriculture as biocontrol agents (Phong et al., 2016; Lang et al., 2012) and/or biostimulants (Spinelli et al., 2022; Yang et al., 2014). An experiment with fungal community of tobacco soil rhizosphere showed that the abundance of *Humicola* was enhanced by the reduction of nitrogen fertilization, while the abundance of *Chaetomium* decreased (Shen et al., 2022). In this study, the relative abundance of these two genera was not significantly correlated with nitrogen compounds (NO_3_-N and NH_4_-N). The prevalence of both genera in sampling sites from cropping system H indicated that the soil management practices, mainly the input of nitrogen fertilizers (organic or inorganic) is suitable to the growth of these beneficial fungi.

Fungal communities from cropping system V are more even than from H. The genera *Penicillium* (Aspergillaceae) and *Humicola* stand out with higher relative abundances. *Penicillium* was identified as a bioindicator for V. This genus is commonly found in soil samples (Wahome et al., 2023; Jamil et al., 2023; Hernandez and Menéndez, 2019) and is associated to the reduction or suppression of soil-borne plant pathogens, due to their capacity to produce compounds with antifungal activity (Puig and Cumagun, 2019; Win et al., 2021). In addition, some species have demonstrated the ability to solubilize phosphate and enhance the plant development (Sharma et al., 2013; Radhakrishnan et al., 2014). Hernandez and Menéndez (Hernandez and Menéndez, 2019) found that species of *Penicillium* were specific to concrete environmental conditions (season and weather) and soil treatments (tillage/no-tillage, herbicide/no-herbicide) in soils from vineyards. In our study, the genus *Penicillium* was found in higher abundance in cropping system V and in lower abundance in B, which may indicate that some species of *Penicillium* are vulnerable to management practices, mainly the input of nutrients, used in B and at less extent in cropping system H. The results indicate that high concentrations of P and Fe are correlated to a decrease in abundance of this genus. Gobbi et al. (2022) found that *Fusarium* was dominant with a relative abundance of up to 10% of total fungal community in vineyard soils from Portugal (Azores). However, in our study, *Fusarium* did not appear in the top 35 genera and the Nectriaceae family to which it belongs represented only about 1% of total community in vineyards. This may be due to the climatic differences between the two Portuguese archipelagos (Pinheiro de Carvalho et al., 2022; Macedo et al., 2020).

The saprotrophs were the trophic group dominating in all three cropping systems. Saprotrophs are the most common functional group in agricultural soils (Camacho-Sanchez et al., 2023; Schmidt et al., 2019; Moreno et al., 2021). The saprotrophs dominance indicates that the soils under study are, in general, rich of fungi capable to provide important agroecological services. Some examples of those fungi were already discussed above. The cropping system B showed lower abundance of some groups of saprotrophs and mycorrhizal fungi but higher abundance of endophytes. The changes in relative abundance of functional groups were well correlated with soil physicochemical properties. The content of N, P, K, and Fe and level of CEC, are influencing the changes observed. Banana requires high amounts of N, P, and K to obtain good yields (Al-Harthi and Al-Yahyai, 2009). For many years, producers of banana from Madeira applied NPK fertilizers (mainly, N – 13%, P – 13%, K – 21%) two to four times/year. Although this practice is currently discouraged, soils accumulated large amounts of these macronutrients over time. Most of the banana agrosystems in this study are established for more than 50 years, thus explaining the higher concentrations of N, P, and K comparing to soils from V and H. In addition, it is a common practice to deposit leaves and pseudostem of banana plants in the soil, returning part of the absorbed nutrients into the soil, including Fe (Ho et al., 2015; Zou et al., 2022). Plant waste from vines and vegetables are likewise incorporated in the soil. However, this occurs in smaller amounts and, in some cases the plant waste can be used for other purposes, such as animal feeding, thus fewer mineral nutrients returned to the soil. Although important functional groups were negatively impacted, these agricultural practices favored the endophytes, fungi that are capable of colonize internal tissues of plants without causing damage and provide important benefits to the plants (Kuźniar et al., 2019). For example, they may stimulate the plants growth (Ozimek and Hanaka, 2021), enhance the host defense efficiency (Cui et al., 2017), and facilitate nutrient absorption (Hiruma et al., 2016). Nevertheless, endophytic fungi may also develop a commensal or pathogenic relationship with the host, under specific conditions (Hiruma et al., 2016; Alam et al., 2021; Cui et al., 2021).

## 5 Conclusion

Our results showed that although alpha-diversity is similar across the three analyzed cropping systems, there are differences in fungal composition and functional characteristics. The dominant phyla were Ascomycota, Basidiomycota, and Mortierellomycota, with variations among the cropping systems from class to downward levels, especially in cropping system B. Agricultural practices adopted in banana plantation from Madeira Island increased P, K, N, and Fe levels while decreasing Cu, influencing the structure of fungal communities. Each cropping system showed an affinity for different fungal genera, highlighting that both soil properties and cultivated crops are key factors in fungal composition.

Overall, the three cropping systems favored the growth of beneficial fungi to the agrosystems, suggesting that local traditional practices are on the right path toward sustainable agriculture.

However, 9% of total ASVs could not be assigned to a phylum, representing around 20% of the community in each cropping system. These unidentified fungi may also have important functions in the agrosystems, reinforcing the need for more studies on agricultural soil fungi to promote sustainable agriculture.

## Data Availability

The data presented in the study are deposited in the Genbank repository (https://www.ncbi.nlm.nih.gov/genbank/), acession number KIGQ00000000.
