# Supplementary material for: Variations in the structure and function of the soil fungal communities in the traditional cropping systems from Madeira Island
Source: Front Microbiol. 2024 Oct 1;15:1426957. doi: 10.3389/fmicb.2024.1426957 (PMC11473422; doi:10.3389/fmicb.2024.1426957)
Supplement: Supplementary file 1 [file Data_Sheet_1.pdf]

Supplementary material

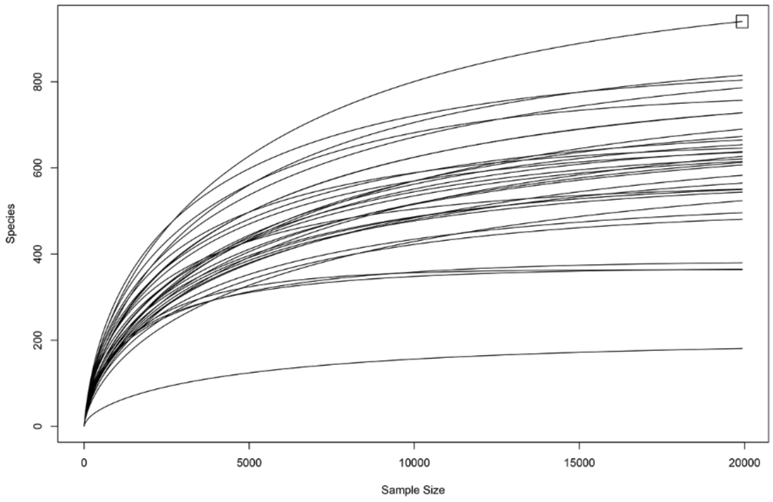

Figure 1 – Rarefaction curves from 18 agrosystems (in duplicates).

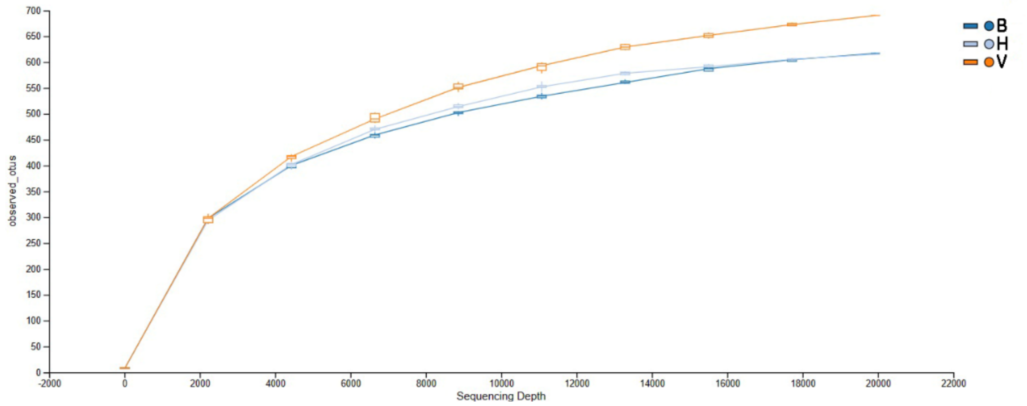

Figure 2 – Rarefaction curves for the three cropping systems (B, V and H).

Table 1 – Average values for diversity indices in cropping systems ± standard error. Mean values and standard error for Richness (Obs. ASVs), Pielou's Evenness (E) and Shannon–Wiener diversity index (H') of the three cropping systems.

Sample	Obs. ASVs	E	H'
**V**	641 ± 113	0.67 ± 0.05	6.23 ± 0.51
**B**	601 ± 71	0.64 ± 0.04	5.90 ± 0.40
**H**	583 ± 83	0.61 ± 0.08	5.65 ± 0.82

Table 2 - Soil physicochemical properties from the 18 agrosystems.

	pH [KCl]	MO [%]	P [ppm]	K [ppm]	BT (Ca) [meq/100g]	BT (Mg) [meq/100g]	BT (Na) [meq/100g]	CEC [meq/100g]	DS [%]	Boro [ppm]	NO_3-N [ppm]	NH_4-N [ppm]	Cu [ppm]	Zn [ppm]	Mn [ppm]	Fe [ppm]
**V1**	5,6	5,07	550	600	20,4	6,4	0,3	32,9	88	0,6	10	2,2	40	11	17,5	40
**V2**	4,5	3,19	137	600	8,3	1,6	0,3	22,4	54	0,6	10	2,1	30	9	18,5	35
**V3**	5,6	2,97	595	1080	17,2	6	0,6	46,1	57	0,5	65	3,5	20	10,5	19	45
**V4**	4,3	7,85	137	480	5,1	1,2	0,3	46,1	18	0,6	30	4,3	45	6	10	35
**V5**	5,8	3,06	687	960	23	6	0,3	38,2	84	0,6	20	2,6	25	8	13	45
**V6**	4,9	6,03	92	480	7,2	2,5	0,6	43,4	27	0,6	10	2,8	10	3,5	12	45
**B1**	6	5,27	1603	1680	38,7	16,8	0,8	59,2	100	0,6	75	7,2	2	10,5	16,5	50
**B2**	5,1	3,54	1374	1200	12,5	9,9	0,3	67,1	39	0,6	15	2,5	6,5	6	15	55
**B3**	3,3	3,25	1557	1440	7,2	7,3	0,5	46,1	41	0,6	25	3,7	3,5	5	16	55
**B4**	3,6	4,78	779	1080	9,9	6	0,3	93,4	21	0,6	130	5,7	4,5	6	16,5	55
**B5**	3,8	5,65	687	1080	8,8	4,7	0,5	40,8	41	0,6	50	5,6	12	10,5	17	50
**B6**	6,2	2,68	641	1560	30,9	11,6	1	43,4	100	0,6	15	2,7	7	11,5	18	45
**H1**	4,6	3,83	183	540	13,3	5,1	0,2	40,8	50	0,6	40	4,9	3	5	19	45
**H2**	6	6,03	687	600	28,2	6	0,5	35,5	100	0,7	25	3,7	5,5	11,5	16,5	45
**H3**	5,3	3,06	183	348	14,1	6	0,7	56,6	38	0,6	10	2,2	3	6	18,5	35
**H4**	4	7,18	595	840	5,1	1,2	0,2	46,1	20	0,6	40	2,5	50	7	16	45
**H5**	5,2	3,15	687	444	18,8	7,3	0,3	35,5	78	0,5	20	3,4	20	6,5	18	45
**H6**	4,4	9,17	137	408	5,9	1,2	0,3	46,1	19	0,6	45	6,5	15	7,5	11	35
